# A randomized controlled trial to assess the effectiveness and safety of acupuncture for overactive bladder: a study in Hong Kong population

**DOI:** 10.1186/s13020-020-00388-w

**Published:** 2020-10-08

**Authors:** Zhi-xiu Lin, Ngai Ho Tony Chan, Yiu-keung Kwan, Yu Tat Chan, Hongwei Zhang, Kam-yuk Sylvia Tam, Mei Kwan Lai, Chun-Kam Lee, Kit Ngan, Stella Yin Yuen Tsoi, Yiu Wa Lau, Yan-Fang Xian, Jessica Ching, Yuanqi Guo

**Affiliations:** 1 School of Chinese Medicine, Faculty of Medicine, The Chinese University of Hong Kong, Shatin, N.T., Hong Kong SAR China; 2 Department of Medicine & Geriatrics, Pok Oi Hospital, Yuen Long, N.T., Hong Kong SAR China; 3 Department of Medicine & Geriatrics, Tuen Mun Hospital, Tuen Mun, N.T., Hong Kong SAR China; 4 Yan Oi Tong – The Chinese University of Hong Kong Chinese Medicine Centre for Training & Research, Tuen Mun, N.T., Hong Kong, SAR China; 5 Hong Kong Institute of Integrative Medicine, The Chinese University of Hong Kong, Shatin, N.T., Hong Kong SAR China; 6 Pok Oi Hospital – The Chinese University of Hong Kong Chinese Medicine Centre for Training & Research, Shatin, N.T., Hong Kong SAR China

**Keywords:** Acupuncture, Overactive bladder, Hong Kong population, Randomized controlled trial, Effectiveness, Safety

## Abstract

**Background:**

Around 15% of the Hong Kong population was found to suffer from overactive bladder (OAB), but the current available treatments, such as medication, behavioral therapy and physical therapy are unsatisfactory. Previous studies have suggested that acupuncture may have promising effect for OAB, but some limitations on the study design render the evidence questionable. This study aimed to evaluate the effectiveness and safety of acupuncture treatment for patients with OAB in Hong Kong.

**Methods:**

One hundred patients with OAB were enrolled. The patients were randomized to receive either active acupuncture or sham needle intervention twice a week for 8 consecutive weeks, and had a follow-up consultation 12 weeks after the completion of acupuncture intervention. The primary outcome assessment was the 3-Day Voiding Diary, which records daytime and night-time urinary frequency and symptoms, at the baseline, the end of the 8-week intervention and 12 weeks after acupuncture intervention. Secondary outcomes included Urine NGF level, Incontinence Impact Questionnaire (IIQ-7) and Urogenital Distress Inventory (UDI-6), as well as Overactive Bladder Symptom Score (OABSS).

**Results:**

After 16 sessions of treatment, when compared with the baseline, both active and sham acupuncture significantly reduced the frequency of urgency urinary incontinence (UUI), daytime and night-time urinary frequency as well as the scores of IIQ-7, UDI-6 and OABSS. Moreover, the treatment effects could last for at least 3 months. However, no significant difference in frequency of UUI and daytime urinary frequency was found between the active and sham acupuncture groups. On the other hand, the night-time urinary frequency decreased more significantly during the treatment and follow-up in the active acupuncture group than in the sham control group after controlling baseline night-time urinary frequency. Urine NGF level could not be detected by ELISA method in our experiments.

**Conclusion:**

This study suggests a beneficial effect of acupuncture on improving OAB symptoms. Both active and sham acupuncture treatment were able to improve the symptoms of frequency of urgency urinary incontinence, and the daytime and night-time urinary frequency, while only mild adverse effects were found. This project was unable to establish the specific effect of acupuncture for OAB.

*Trial registration* Chinese Clinical Trial Registry, ChiCTR-INR-16010048. Registered on 29 Nov 2016.

## Background

Overactive Bladder, also known as OAB, is defined as “urgency, with or without urge incontinence, usually with frequency and nocturia” by the International Continence Society (ICS) [1]. OAB is usually categorized into two different types, i.e. OAB dry and OAB wet type according to the clinical symptoms. OAB dry is defined as having ≧ 4 episodes of urgency in the previous 4 weeks, with either frequency > 8 times per day or the use of ≧ 1 coping behavior to control bladder function. OAB wet meets all the criteria of the OAB dry, but also have ≧ 3 episodes of incontinence in the past 4 weeks that are clearly not caused by stress incontinence [1].

The prevalence of OAB increases with age for both sexes, and it was estimated to be 2.1% in a population older than 40 years in China in 2011, of whom 1.0% had OAB dry and 1.1% had OAB wet. The prevalence of OAB was more common in men than in women over the age of 60 (4.6% vs. 2.6%) [2]. OAB prevalence was estimated to be 4.41% in a cohort study of women over 18 years old in the USA in 2016 [3]. According to the World Population Aging 2019, the population aged 60 or over in Hong Kong reached 17.5% of the total population in 2019 [4]. With the pace of population aging gathering speed, it is expected that there will be more OAB patients in Hong Kong in the coming decades.

The medical expenditure for OAB is enormous. The total medical costs for patients with OAB of six industrialized countries, including Canada, Germany, Italy, Spain, Sweden and the UK, have been estimated to be approximately €3.9 billion annually [5]. In a recent study in Hong Kong, around 15% of population was found to suffer from OAB [6]. The economic burden resulting from managing OAB in Hong Kong will be huge in the foreseeable future. Moreover, OAB can also lead to impaired quality of life to the patients [7].

The current treatment methods for OAB include pharmacological, behavioral and physical therapies. Unfortunately, the outcomes of these methods are largely unsatisfactory. Medication is one of the most commonly used treatment methods for OAB in Hong Kong. Presently, anticholinergics remain the mainstay of therapeutic drugs for OAB; however, it is only partially effective. Moreover, these drugs have considerable side effects such as dry mouth, dry eye, cessation of perspiration or even photophobia or confusion. On the other hand, a number of studies have reported that behavioral therapy and physical therapy are also effective; however, their treatment effects are not sustainable, and usually decline 3 month after treatment [7].

In mainland China as well as in Hong Kong, patients with OAB often seek help from acupuncture treatment. Acupuncture is one of the most widely practiced alternative and complementary treatment modalities in East Asia and the Pacific Region. Moreover, a few clinical trials have been conducted to evaluate the effectiveness and the safety of acupuncture for the treatment of OAB [8]. A pilot study conducted by Engberg et al. [9] suggested that acupuncture may have clinical meaningful effect on urge incontinence. A clinical case report (n = 11) by Kitakoji et al. [10] indicated that acupuncture may elicit an improvement on OAB symptoms. A review of 10 randomized controlled trials (RCTs) with 794 patients reported that acupuncture might have effect in decreasing the number of micturition, incontinence, and nocturia episodes. However, the available evidence is insufficient to support the effect of acupuncture alone or its additional effect to drugs in treating OAB [8]. Several limitations of these studies may potentially bias the result and render the findings inconclusive. Firstly, many studies were open label trials or single blinding, i.e. with blinding not applied to patients or outcome assessors [5, 11, 12]. Secondly, some did not use control arm. Thirdly, the sample size of many trials was generally small [5, 12]. Fourthly, many studies used the technique of sham acupuncture which involved the penetration of skin on the non-specific acupuncture points which the authors believed would not produce treatment effect [12]. However, it has been well-known that any acupuncture involving penetration of the skin, regardless on the acupuncture points or not, would elicit physiologic response, hence treatment effect [13]. Fifthly, most of these studies focused only on female sufferers, and few evaluated efficacy of acupuncture in male OAB sufferers [8].

On a local perspective, there had not been any study conducted in Hong Kong to investigate the effectiveness and safety of traditional acupuncture treatment for OAB. There clearly existed a need to fill this knowledge gap. Based on the above discussion, a full-scale randomized controlled trial was proposed with an aim to establish clinical evidence about the effectiveness and safety of acupuncture for treating OAB patients in Hong Kong.

## Methods

### Study design

We undertook a 20-week randomized, patient and outcome assessor blinded, sham-controlled trial to assess whether the acupuncture could ameliorate the subjective symptoms in OAB patients.

During the study, a total of 100 OAB patients were successfully recruited. The subjects were asked to discontinue any active anti-OAB treatment such as anticholinergics, or diuretic medication, and each patient received a total of 16 sessions of acupuncture (active or sham treatment) over 8 weeks. The 3-day voiding diary was used as the primary outcome measure.

All patients provided written informed consent prior to the study, and the study was conducted at the CUHK Chinese Medicine Specialty Clinic cum Clinical Teaching and Research Centre (CUHK-CMSCcCTRC) at the School of Chinese Medicine, CUHK and the Yan Oi Tong—The Chinese University of Hong Kong Chinese Medicine Centre for Training and Research in Tuen Mun (YOT-CUHKCMCTR). The study protocol was approved by the Joint CUHK-NTEC Clinical Research Ethics Committee (CREC Ref. No.: 2017.199-T) and the NTWC Clinical Research Ethics Committee (CREC Ref. No. NTWC/CREC/15,147). The trial was conducted according to the principles of Good Clinical Practice and the Declaration of Helsinki.

### Trial participants

Eligibility criteria were: (1) men or women aged between 60 to 90 years old; (2) diagnosed as OAB according to the diagnosis criteria of the ICS; and (3) physically and mentally able to complete the 3-day voiding diary, Urinary Distress Inventory (UDI-6) and Incontinence Impact Questionnaire (IIQ7); and (4) able to give written informed consent (by patients themselves or by their caretakers).

The exclusion criteria included (1) OAB symptom caused by stroke or spinal injury; (2) life threatening infection; (3) unconsciousness or severe cognitive deficits; (4) dementia caused by Alzheimer’s disease or other neurodegenerative diseases; (5) undergone incontinence surgery previously; (6) on short-term active diuretic treatment or taking diuretic medication; (7) had received acupuncture treatment for OAB within 2 months prior to the study; (8) pregnancy; and (9) suffering from the following diseases: untreated urinary tract infection, urogenital tumors, prostate tumor, benign prostatic hyperplasia, chronic urinary retention. Anyone who met all the inclusion criteria and had none of exclusion criteria was eligible for this study.

### Randomization and blinding

The block randomization was used to generate random allocation numbers through a computer program. The list of randomization number was kept by a research staff member who was responsible for assigning the corresponding intervention code based on the list to the recruited patients. He was not involved in patient care, outcome assessment, data collection or data analysis. Included participants were randomly allocated to either active acupuncture group (also called intervention group) or sham acupuncture group (also called placebo-control group) after the baseline assessment. All the participants, outcome assessors and clinical investigators were kept blind during the whole study. But owing to the manual manipulation nature of acupuncture, it was not possible to blind the acupuncturists who manage the treatment procedure. However, they were instructed to keep minimum interaction with patients during the treatment period. The blinding code was not opened during the study period.

To test the success of masking, a direct question was asked to each participant: “Before the study was conducted, you were informed that you have equal chance to receive real or sham acupuncture treatment. After that, which treatment do you think you have received?” Three answers were provided: real acupuncture, sham acupuncture, and unknown.

### Interventions

The participants assigned to the intervention group received, in addition to standard care, standardized 30-min acupuncture treatment sessions. According to the traditional Chinese medicine theory, the pathogenesis of overactive bladder symptoms is mainly attributed to insecurity of kidney qi (腎氣不固). Based on the traditional acupuncture theory, previous relevant studies, and opinion of acupuncture experts of the research team, the following acupuncture points were selected for treatment: BL32 (Ciliao, 次髎) (bilateral), BL23 (Shenshu, 腎俞) (bilateral), SP6 (Sanyinjiao, 三陰交) (bilateral), KI3 (Taixi, 太溪) (bilateral), BL39 (Weiyang, 委陽) (bilateral), BL28 (Pangguangshu, 膀胱俞) (bilateral), and CV4 (Guanyuan, 關元). The locations, therapeutic indications and manipulations of the acupuncture points selected in this study have been shown in our previously published research protocol [14].

To ensure the blinding effectiveness to patients, the sham acupuncture used in this study was a type of acupuncture in which needles with the same size and shape as those of intervention group, but with the needle tips made blunt. Moreover, the sham needles were not penetrated into the skin with the blunt tips; instead, the needles were retracted into needle handles, thus preventing penetration through the skin at the same points as the treatment group. Therefore, the subjects in the sham control group would be unlikely to see whether the needles were penetrated into the skin or not.

The acupuncture treatment was administered twice per week, which mimics the clinical practice of using acupuncture treatment for OAB in many Chinese medicine clinics in Hong Kong, and the whole treatment period lasted for 8 consecutive weeks. A total of 16 sessions of acupuncture treatment was administered to each participant. The sham acupuncture treatment was administered on the same acupuncture points with sham needles, while the treatment procedure of sham acupuncture mimicked that of real acupuncture in the whole study.

All acupuncture treatments were carried out by Registered Chinese Medicine Practitioners who had at least 3 years of clinical experience in acupuncture practice. Moreover, the acupuncturists involved in the study were trained by the acupuncture experts of the research team to ensure the consistency of the protocol of the acupuncture treatment.

As an incentive measure for patient compliance, for patients who were assigned to sham acupuncture group, we provided free acupuncture treatment for their OAB after they had completed the entire study including the 3-month follow-up period.

### Data collection

Outcome measurements at baseline, the end of treatment (2 months after inclusion) and at the follow-up period (3 months after treatment completion) were conducted. All data were collected and stored safely for further analysis.

### Primary outcome measure

Among these variables, the primary outcome measurement was the reduction in the frequency of urgency urinary incontinence (UUI) as derived from the 3-day bladder diaries [12]. Essentially, the 3-Day Voiding Diary records daily urination and symptoms such as the time of urination and frequency of UUI in the most recent three days. The 3-Day Voiding Diary had the following advantages: (a) the validity of the scale has been proved by previous study; (b) it is a non-invasive assessment tool which minimizes the risk and inconvenience of the patient; and (c) the diary in Chinese version has been validated and readily available for use. The copy of the voiding diary is shown in Appendix 1.

The outcomes recommended by ICS, such as OABSS (see Appendix 2), IIQ-7 and UDI-6 (see Appendix 3) were used as secondary outcome measures [15]. All of the IIQ-7, UDI-6 and OABSS chosen in this study were available in Chinese version, which were known to be as sensitive, valid and reliable as original version and other languages [16].

### Tolerability and safety evaluation

All adverse events as defined by the Ethics Committee will be fully recorded on the Adverse Event Page of the CRF. If any serious adverse effect occurred, emergency medical assistance would be required. All serious adverse effects were recorded in SAE report form. Documentation was supported by an entry in the participant’s medical record. Signs and symptoms of each adverse event would be described in detail: date of onset, intensity, outcome, date of resolution, and any action taken. The PA was ultimately responsible for promptly notifying the Ethics Committee of all serious adverse events, including follow-up information.

### Statistical analysis including sample size estimation

The primary outcome measure is the reduction in the frequency of UUI as derived from the 3-day voiding diary. Based on a previous study of Emmons and Otto [12], the placebo effect was 40% reduction in frequency of UUI among patients treated with placebo acupuncture. To detect a clinically meaningful effect of 30% difference in the change of frequency of UUI between the treatment and placebo control group, 41 cases will be needed in each group to have 80% power to detect difference between the two groups at the significance level of 0.05. To allow for a predicted dropout rate of 20%, a total of 100 patients will be required for this study, with 50 patients in each arm.

Descriptive statistics was computed for each of the analyzed variables. The baseline characteristics were tabulated. The statistically significant differences between the two groups were tested by independent t test for continuous data, chi-square test for frequency data, and Mann–Whitney test for frequency of UUI. The generalized linear model was used to compare primary and secondary outcomes between the two groups with controlling baseline night-time urinary frequency. Per-protocol analysis was conducted, and only patients who strictly followed the protocol and completed the study at each time point were included in the analysis. All statistical tests were two-sided, and *p* < 0.05 was considered statistically significant. The SAS software (9.4 version) was used for the analysis.

## Results

### Study flow

**Fig. 1:**
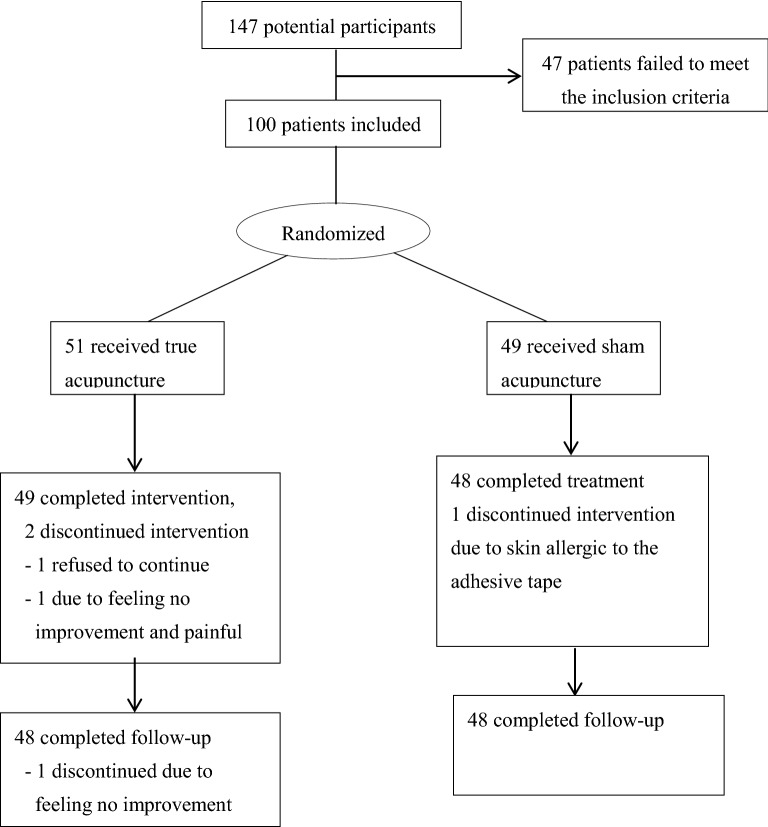
Study flow of the trial

A total of 100 patients with OAB were successfully recruited during the period between June 2016 and September 2019 in the CUHK-CMSCCTRC or YOT-CUHKCMCTR. Three promotion articles were posted on the Sky Post in 09/2017, 04/2018 and 11/2018 to augment subject recruitment. We screened 147 participants, and 47 were excluded because they failed to meet the Inclusion criteria. After random allocation, 51 patients received active acupuncture and 49 received sham acupuncture intervention. Totally 4 participants withdrew from the study; among them, 2 participants in the treatment group withdrew before the end of treatment, among whom 1 complained of feeling no improvement and pain during acupuncture treatment and another refused to continue without explanation. In addition, 1 participant in the treatment group was lost to follow up due to feeling no improvement. One participant in the sham control group withdrew before the end of intervention due to skin allergic to the adhesive tape. Figure 1 shows the study flow and reasons for drop-out.

### Test of the effectiveness of blinding

We obtained blind evaluation data from 93 out of 100 participants. 97.9% of patients in the real acupuncture group guessed right, and 80.4% of those in the sham group thought they had received real acupuncture or were not sure about the choice.

We calculated the blinding index (BI) as proposed by Bang et al. [17]. Bang’s BI is interpreted as the percentage of unblinding beyond chance. It takes a value between -1 and 1, where 1 corresponds to all correct guesses, whereas − 1 corresponds to all incorrect or opposite guesses. If 50% of patient responses are correct and 50% are incorrect, then BI = 0; this is indicative of random guessing and thus is an ideal blinding scenario. Generally, it is considered as BI ≥ 0.2 for unblinded; − 0.2 < BI < 0.2 for random guesses, and BI ≤  − 0.2 for opposite guesses [17].

Bang’s BI in the real acupuncture group is 0.96 (95% CI 0.87 to 1.04), and in the sham control group is − 0.59 (95% CI − 0.82 to − 0.36). The result indicated the attainment of effective blinding, where patients tend to believe they received active treatment regardless of actual treatment received, which may reflect patients’ wish to receive active intervention. This finding is consistent with the results of a systematic review of acupuncture trials, which reported the most common scenario in the review was unblinded in the acupuncture group and opposite guess in the sham acupuncture group. Such results could be indeed interpreted as “well-blinded” [18]. It should be noted that in this scenario there may be a psychological phenomenon of “wishful thinking”, and guesses are inflated towards real acupuncture in both groups.

### Baseline characteristics

Table 1 shows the baseline characteristics and assessments of the participants in both treatment and control groups. The mean age of the 100 patients was 68.5, and 55 of them (55%) were women. The baseline characteristics and assessments did not differ significantly between the two groups except night-time urinary frequency. The mean night-time urinary frequency was markedly higher in the treatment group (8.08 ± 4.66) than in the control group (5.73 ± 3.54).

**Table 1:** Baseline characteristics of the participants in both treatment and control groups

	Treatment group (n = 51)	Control group (n = 49)	P value
Age (years)			
Mean ± SD	68.96 ± 6.75	67.94 ± 5.65	0.42
60–69, n (%)	33 (64.7)	32 (65.3)	
70–79, n (%)	14 (27.5)	15 (30.6)	
80–89, n (%)	3 (5.9)	2 (4.1)	
90, n (%)	1 (1)	0 (0)	
Gender			
Male, n (%)	19 (37.3)	26 (53.1)	0.16
Female, n (%)	32 (62.7)	23 (46.9)	
3-day voiding diary, mean ± SD			
All-day urinary frequency	42.60 ± 11.24	41.71 ± 11.27	0.70
Daytime urinary frequency	33.90 ± 11.44	36.57 ± 9.73	0.21
Night-time urinary frequency	8.08 ± 4.66	5.73 ± 3.54	0.006*
Frequency of UUI, median (range)	8.66 (0–54)	7.10 (1–44)	0.16
25 percentile	2	2	
75 percentile	10	7	
IIQ7	44.44 ± 25.80	49.00 ± 25.86	0.38
UDI6	39.95 ± 13.62	39.54 ± 14.06	0.88
OABSS	10.25 ± 2.17	10.33 ± 2.25	0.87

Statistical significance was tested by using independent t test for continuous data, chi-square test for frequency data, and Mann–Whitney test for frequency of UUI

^*^ *p* < 0.05

IIQ: Incontinence Impact Questionnaire, Short Form. UDI6: Urinary Distress Inventory, Short Form. OABSS: Overactive Bladder Symptom Score

### Primary outcome

**Fig. 2:**
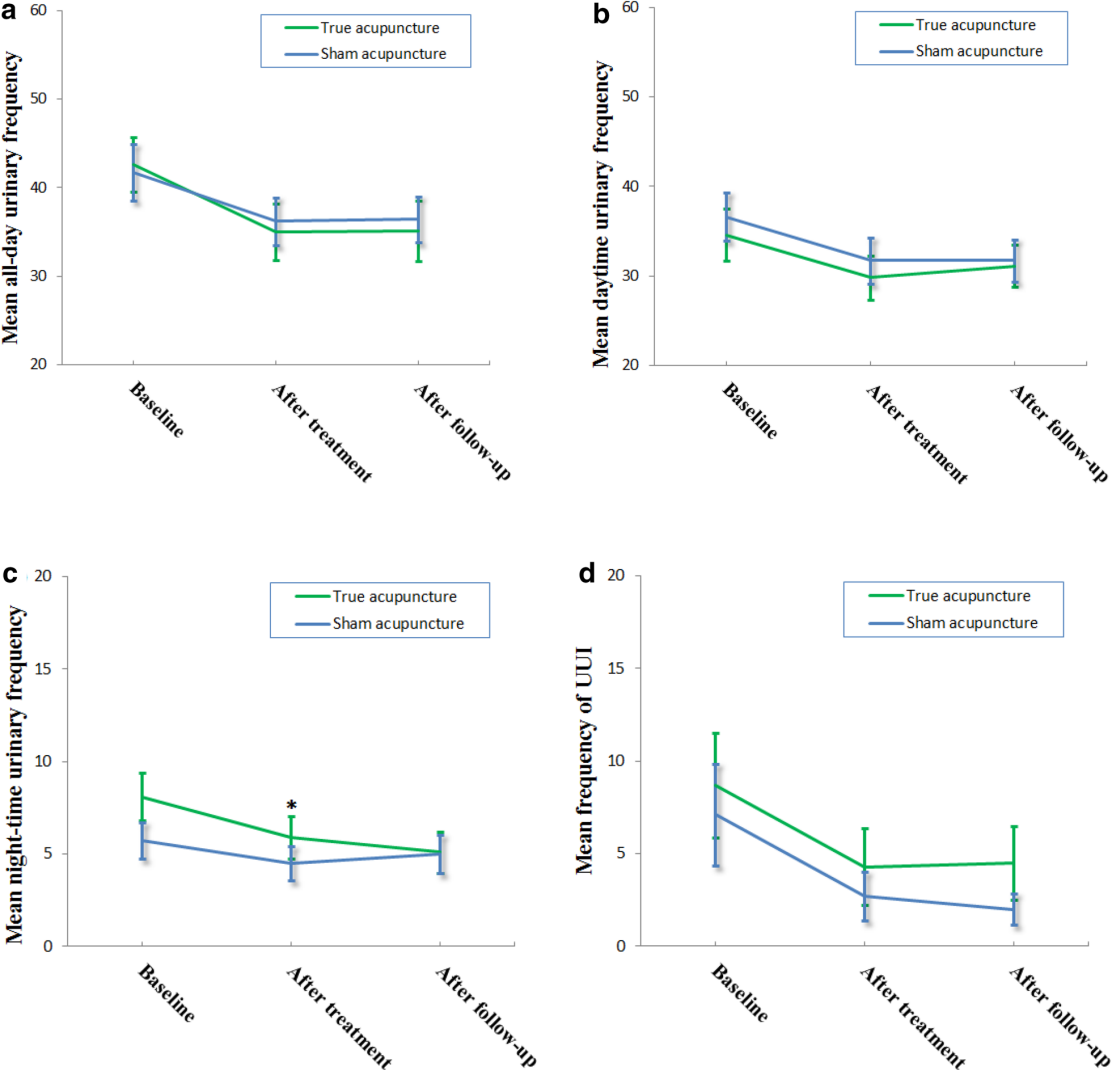
Line charts of all-day, daytime and night-time urinary frequency, and frequency of UUI with 95% CI in the treatment and control group. NB The differences between baseline and after treatment in all-day, daytime and night-time urinary frequency, and frequency of UUI in true and sham acupuncture group are statistically significant. * There is a statistically significant difference in night-time urinary frequency between the two groups

Table 2 and Fig. 2 show the daytime and night-time urinary frequency and frequency of UUI recorded in the 3-Day Voiding Diary in both groups. The frequency of UUI at the completion of the active or sham acupuncture treatment, and at the follow-up decreased significantly when compared to the baseline in both groups (*p* = 0.0016) (Table 3). However, no significant difference in frequency of UUI after the treatment was found between the two groups after controlling baseline night-time urinary frequency (*p* = 0.75) (Table 3).

**Table 2:** The 3-day voiding diary during the trial (mean (SD))

	True acupuncture	N1	Sham acupuncture	N2
All-day urinary frequency				
Baseline	42.6 (11.2)	50	41.7 (11.3)	49
After treatment	35.0 (11.2)	48	36.2 (9.5)	48
At follow-up	35.1 (11.9)	47	36.4 (9.0)	48
Daytime				
Baseline	34.6 (10.5)	50	36.6 (9.7)	49
After treatment	29.8 (8.7)	48	31.7 (9.2)	48
At follow-up	31.1 (8.2)	47	31.7 (8.4)	48
Night-time				
Baseline	8.1 (4.7)	50	5.7 (3.5)	49
After treatment	5.9 (4.1)	48	4.5 (3.3)	48
At follow-up	5.1 (3.9)	47	5.0 (3.6)	48
Frequency of UUI				
Baseline	8.6 (10.3)	50	7.1 (9.7)	49
After treatment	4.3 (7.4)	48	2.7 (4.6)	48
At follow-up	4.5 (6.9)	47	2.0 (3.0)	48
Frequency of UUI Median (25, 75 percentile)				
Baseline	5 (2, 10)	50	4 (2, 7)	49
After treatment	1 (0, 5.8)	48	1 (0, 3.8)	48
At follow-up	2 (0, 7)	47	1 (0. 3)	48

**Table 3:** Effect of group with regard to time on the frequency of UUI by generalized linear model

Source variable: Frequency of UUI	df	F	p-value
time	2	6.82	0.0016
time*group	2	0.29	0.7463
time*age	40	0.56	0.9812
time*gender	2	0.09	0.9161
time*night0_urine	30	0.83	0.7111

Daytime and night-time urinary frequency at the completion of the acupuncture treatment decreased significantly when compared to the baseline in both groups. The night-time urinary frequency decreased more significantly during the treatment and follow-up in the active acupuncture group than in the sham control group after controlling baseline night-time urinary frequency (*p* = 0.028) (Fig. 2, Table 4). No significant difference was found in the daytime urinary frequency after treatment and follow-up between the two groups.

**Table 4:** Effect of group with regard to time on night-time urinary frequency by generalized linear model

Source variable: Night-time urinary frequency	df	F	p-value
time	2	17.24	< .0001
time*group	2	3.66	0.0288
time*age	40	1.56	0.037
time*gender	2	2.86	0.0615
time*night0_urine	30	2.79	< .0001

### Secondary outcomes

**Fig. 3:**
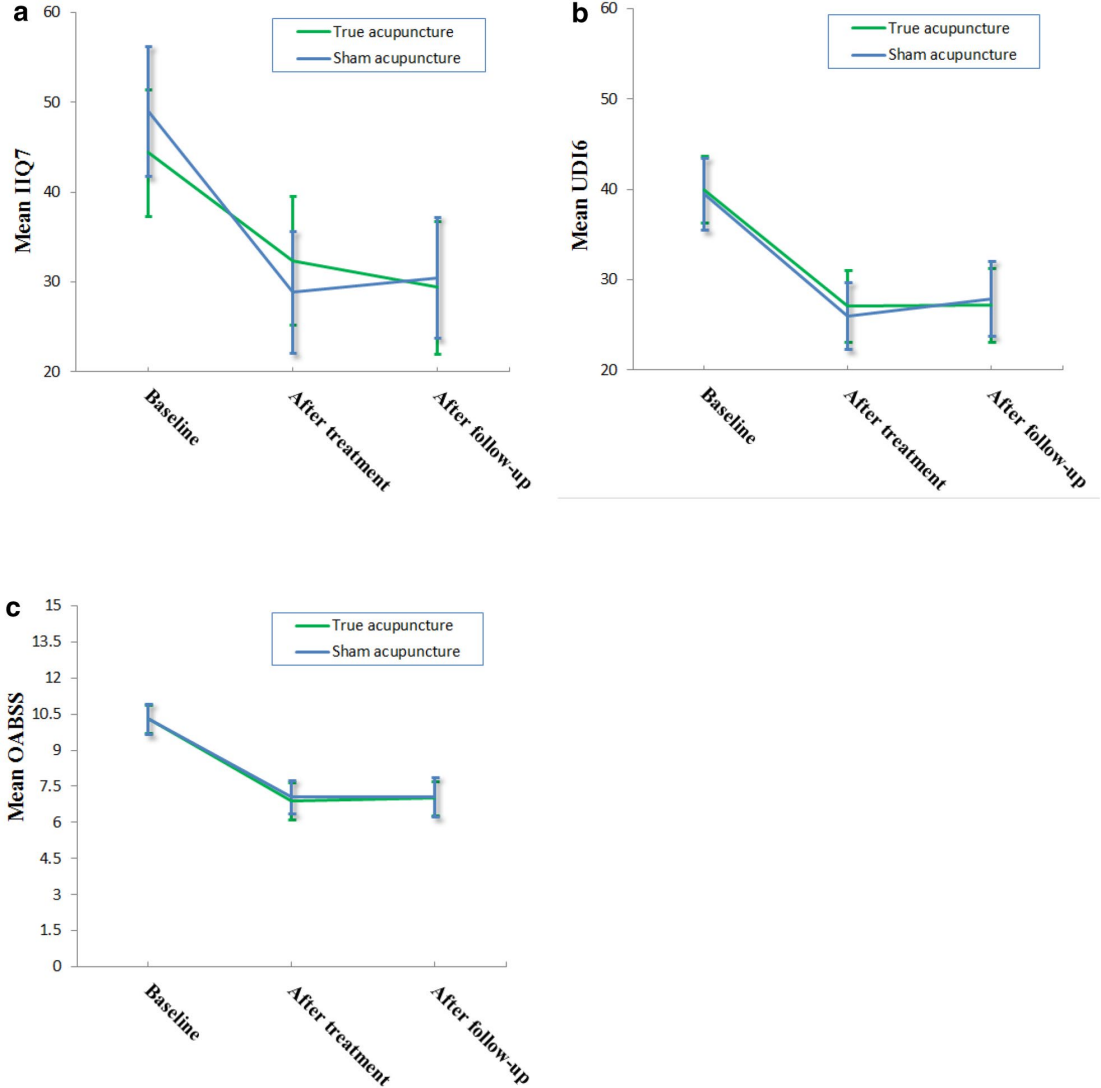
Line charts of IIQ7, UDI6 and OABSS with 95% CI in the treatment and sham control group. NB The differences between baseline and after treatment in IIQ7, UDI6 and OABSS in true and sham acupuncture group are statistically significant

Table 5 and Fig. 3 show IIQ-7, UDI-6 (including their subscales) and OABSS in the acupuncture and sham control groups. The scores after treatment and follow-up were significantly decreased when compared to the baseline in both groups. No significant difference was found in IQ-7, UDI-6 and OABSS between the two groups after treatment and follow-up (Tables 6, 7 and 8).

**Table 5:** IIQ-7, UDI-6 and OABSS during the trial (mean (SD))

	True acupuncture	N1	Sham acupuncture	N2
IIQ-7 total				
Baseline	44.4 (25.8)	51	49.0 (25.9)	49
After treatment	32.4 (25.4)	49	28.9 (23.8)	48
At follow-up	29.4 (26.2)	48	30.5 (24.9)	48
Physical activity				
Baseline	36.9 (32.5)		44.6 (30.9)	
After treatment	28.6 (27.6)		26.0 (24.5)	
At follow-up	27.1 (28.1)		30.2 (26.1)	
Travel				
Baseline	47.1 (29.8)		52.4 (27.8)	
After treatment	32.3 (29.1)		33.0 (27.8)	
At follow-up	32.6 (27.9)		32.6 (30.2)	
Social/relationship				
Baseline	41.8 (32.6)		55.1 (30.8)	
After treatment	36.7 (30.6)		33.3 (28.4)	
At follow-up	28.5 (30.0)		33.3 (30.8)	
Emotional health				
Baseline	50.7 (27.5)		47.0 (31.1)	
After treatment	34.0 (30.6)		25.3 (26.6)	
At follow-up	30.9 (30.6)		29.9 (28.3)	
UDI-6 total				
Baseline	40.0 (13.6)	51	39.5 (14.1)	49
After treatment	27.1 (14.2)	49	26.0 (13.1)	48
At follow-up	27.2 (14.6)	48	27.9 (14.7)	48
Irritative symptoms				
Baseline	53.2 (13.9)		55.4 (16.1)	
After treatment	39.8 (18.7)		37.2 (18.3)	
At follow-up	38.8 (18.1)		40.9 (19.6)	
Stress symptoms				
Baseline	42.9 (19.2)		40.1 (21.0)	
After treatment	24.0 (18.9)		25.3 (17.2)	
At follow-up	27.3 (19.1)		26.8 (18.6)	
Obstructive discomfort				
Baseline	23.8 (18.4)		23.2 (17.3)	
After treatment	17.6 (16.3)		15.6 (13.8)	
At follow-up	15.4 (17.2)		15.9 (15.6)	
OABSS				
Baseline	10.3 (2.2)	51	10.3 (2.2)	49
After treatment	6.9 (2.8)	49	7.1 (2.4)	48
At follow-up	7.0 (2.5)	48	7.1 (2.9)	48

**Table 6:** Effect of group with regard to time on IIQ-7 total by generalized linear model

Source variable: IIQ-7 total	df	F	p-value
time	2	16.27	< .0001
time*group	2	0.53	0.5903
time*age	40	0.84	0.7298
time*gender	2	1.02	0.3642
time*night0_urine	30	1.14	0.3089

**Table 7:** Effect of group with regard to time on UDI-6 total by generalized linear model

Source variable: UDI-6 total	df	F	p-value
time	2	23.12	< .0001
time*group	2	0.22	0.8015
time*age	40	0.8	0.7925
time*gender	2	0.13	0.8774
time*night0_urine	30	0.66	0.9022

**Table 8:** Effect of group with regard to time on OABSS by generalized linear model

Source variable: OABSS	df	F	p-value
time	2	27.78	< .0001
time*group	2	0.39	0.6769
time*age	40	0.57	0.9792
time*gender	2	1.22	0.2981
time*night0_urine	30	0.66	0.9088

### Adverse events

In our study, no severe adverse effect as a result of acupuncture was experienced by the participants. Two out of 100 (2%) patients reported adverse reactions, which included mild uncomfortable feeling towards acupuncture treatment and skin allergic to the adhesive tape. These 2 participants with mild AE also withdrew from the study, with reasons being the treatment effect was not strong enough worthy of continuation.

## Discussion

### General findings

This randomized, sham controlled clinical trial aimed to evaluate the effectiveness and safety of acupuncture for OAB. We found that after 16 sessions of treatment, when compared with the baseline, both active and sham acupuncture groups showed significant improvement in the reduction of frequency of UUI, daytime and night-time urinary frequency, and the effect could last for at least 3 months (follow-up). It is worth noting that active acupuncture produced more pronounced improvement in the night-time urinary frequency than that of sham acupuncture group. In addition, the scores of IIQ-7, UDI-6 and OABSS in both groups also decreased significantly after treatment when compared with the baseline, while no difference was found between the two groups after treatment and follow-up.

To sum, this study suggests a beneficial effect of both active and sham acupuncture on improving OAB symptoms. A natural history study on women with OAB has found that a remission was seen in 40%, stable disease in 41.3%, a progression in 6.7% and an improvement in 12% of 386 women with a mean age of 54 years (range: 21–81 years) over a period of 6.5 years [19]. The reduction in OAB symptoms after treatment was supposed to be largely attributable to the acupuncture treatment. However, positive effects were also revealed in the placebo group, which is out of our expectation. Several reasons may explain this observation. First of all, OAB is a chronic disease with fluctuating symptoms influenced by various factors, such as life style, diet including the level of alcohol and caffeine intake, mood and gender especially for those women who had a few natural labors. Therefore it is difficult to measure all the variables in the clinical trial. All these confounding factors render it difficult to test the specific effect of acupuncture for the treatment of OAB. Secondly, it has been reported that placebo acupuncture (the sham needles) could produce about 33–56% placebo effect for OAB patients [2, 12]. In this study, we applied sham acupuncture needles to the true acupuncture points, therefore, it is plausible that the sham acupuncture treatment could also elicit therapeutic effects to the subjects. This may explain why placebo group also exerted significant treatment effect similar to the active acupuncture group. The possible specific acupuncture treatment effect may be too small to be differentiated from the placebo effect in this study.

### Limitations

We found a number of limitations associated with this study. Firstly, the number of participants lost to follow up was 4 out of 100, i.e. a 4% drop-out rate, which is not high; however, the drop-outs may have influence to the validity of study results. Therefore, we used the last observation carried forward (LOCF) method for ITT analysis, and this may increase our confidence in the interpretation of findings to some extent. Secondly, like all acupuncture trials, it was not possible to keep the patients totally blinded to their treatment group with sham needling, especially when we had to leave the needles on the acupuncture points for 30 min [12]; also, some patients had received acupuncture before for other disorders.

The 3-Day Voiding Diary was chosen as the primary outcome measure, instead of a 7-Day Voiding Diary suggested in our original research proposal, because of the fact that (1) many participants in our study found it too difficult to handle the 7-Day Voiding Diary in our pilot test; and (2) The 3-Day Voiding Diary is nearly as accurate as the 7-Day Voiding Diary for assessing incontinence [12].

We also conducted the enzyme-linked immunosorbent assay (ELISA) to measure the urinary UGF level in this study. The urine sample was collected from each participant at baseline and weeks 8 and 20 (follow up). The patient was told to collect his or her first morning urine sample on the day when they came to the clinic. Then the urine sample was stored in refrigerator and sent to our laboratories for ELISA analysis in batch. We used ELISA kits from different manufacturers in order to detect the amount of NGF in the urine samples. However, all these attempts were not successful and the readings from the ELISA kits were too low to be meaningful. We believe the amount of NGF presented in the urine samples was far too traced to be detected by even the very sensitive ELISA kits. It should be noted that we routinely perform ELISA analysis in our laboratories.

### Implication and future perspective

The results from the present study amply indicate that acupuncture treatment (both active and sham needling) could significantly improve the OAB symptoms through reducing frequency of UUI, and daytime and night-time urinary frequency. On the other hand, the active acupuncture treatment in this study resulted in more improvement in night-time urinary frequency than that of sham acupuncture treatment. It is therefore suggested that acupuncture may be an alternative treatment option for patients with OAB.

In the present study, we found that even placebo treatment using sham acupuncture also produced significant treatment response, and it became difficult to establish the specific effect of acupuncture for OAB. We suggest to use different sham acupuncture design in future trial in which pressing blunt needles outside true acupuncture points can be adopted. Besides, we should target on those participants who have no prior experience in receiving acupuncture before. Moreover, objective outcome measures should be used as much as practically possible to minimize the expectation of the subjects for acupuncture treatment [20].

## Conclusions

The study has demonstrated that both active and sham acupuncture treatments possess beneficial therapeutic effect for OAB, especially in relieving the symptoms of frequency of UUI, and the daytime and night-time urinary frequency. The active acupuncture treatment improved night-time urinary frequency more than did the sham acupuncture. We have also demonstrated that acupuncture is a safe treatment modality for OAB patients.

## Data Availability

I agree to share my data and materials.

## Abbreviations

BI: Blinding index
ELISA: Enzyme-linked immunosorbent assay
ICS: International Continence Society
IIQ-7: Incontinence Impact Questionnaire
LOCF: Last observation carried forward
OAB: Overactive bladder
OABSS: Overactive Bladder Symptom Score
RCTs: Randomized controlled trials
UDI-6: Urogenital Distress Inventory
